# Establishing a Unified COVID-19 “Immunome”: Integrating Coronavirus Pathogenesis and Host Immunopathology

**DOI:** 10.3389/fimmu.2020.01642

**Published:** 2020-07-03

**Authors:** Els Wauters, Karin Thevissen, Carine Wouters, Francesca Maria Bosisio, Frederik De Smet, Jan Gunst, Stephanie Humblet-Baron, Diether Lambrechts, Adrian Liston, Patrick Matthys, Johan Neyts, Paul Proost, Birgit Weynand, Joost Wauters, Sabine Tejpar, Abhishek D. Garg

**Affiliations:** ^1^Laboratory of Respiratory Diseases and Thoracic Surgery (BREATHE), Department of Chronic Diseases and Metabolism and Department of Respiratory Diseases, University Hospitals KU Leuven, KU Leuven, Leuven, Belgium; ^2^Centre of Microbial and Plant Genetics (CMPG), KU Leuven, Leuven, Belgium; ^3^Pediatric Immune-Inflammatory Diseases, Laboratory of Adaptive Immunology & Immunobiology, Department of Microbiology and Immunology, Department of Pediatrics, University Hospitals KU Leuven, KU Leuven, Leuven, Belgium; ^4^Member of the European Reference Network for Rare Immunodeficiency, Autoinflammatory and Autoimmune Diseases, Leuven, Belgium; ^5^Laboratory for Translational Cell and Tissue Research, Department of Imaging and Pathology, Department of Pathology, University Hospitals of Leuven, KU Leuven, Leuven, Belgium; ^6^The Laboratory for Precision Cancer Medicine, Translational Cell and Tissue Research Unit, Department of Imaging and Pathology, KU Leuven, Leuven, Belgium; ^7^Clinical Department and Laboratory of Intensive Care Medicine, Department of Cellular and Molecular Medicine, KU Leuven, Leuven, Belgium; ^8^Laboratory of Adaptive Immunology, Department of Microbiology, Immunology and Transplantation, KU Leuven, Leuven, Belgium; ^9^Laboratory for Translational Genetics, Department of Human Genetics, VIB Center for Cancer Biology, VIB and KU Leuven, Leuven, Belgium; ^10^Department of Microbiology and Immunology, VIB Center for Brain and Disease Research, The Babraham Institute, Babraham Research Campus, KU Leuven, Cambridge, United Kingdom; ^11^Laboratory of Immunobiology, Department of Microbiology, Immunology and Transplantation, Rega Institute, KU Leuven, Leuven, Belgium; ^12^KU Leuven Department of Microbiology and Immunology, Rega Institute for Medical Research, Leuven, Belgium; ^13^Laboratory of Molecular Immunology, Department of Microbiology, Immunology and Transplantation, Rega Institute, KU Leuven, Leuven, Belgium; ^14^Department of Pathology, University Hospitals KU Leuven, Leuven, Belgium; ^15^Laboratory for Clinical Infectious and Inflammatory Diseases, Medical Intensive Care Unit, University Hospitals KU Leuven, KU Leuven, Leuven, Belgium; ^16^Laboratory for Molecular Digestive Oncology, Department of Oncology, KU Leuven, Leuven, Belgium; ^17^Laboratory for Cell Stress & Immunity (CSI), Department of Cellular & Molecular Medicine, KU Leuven, Leuven, Belgium

**Keywords:** T-cell exhaustion, cytokine storm, macrophages, B-cells, SARS coronavirus, interferons (IFNs), biomarkers, neutrophils

## Introduction

**Figure 1:**
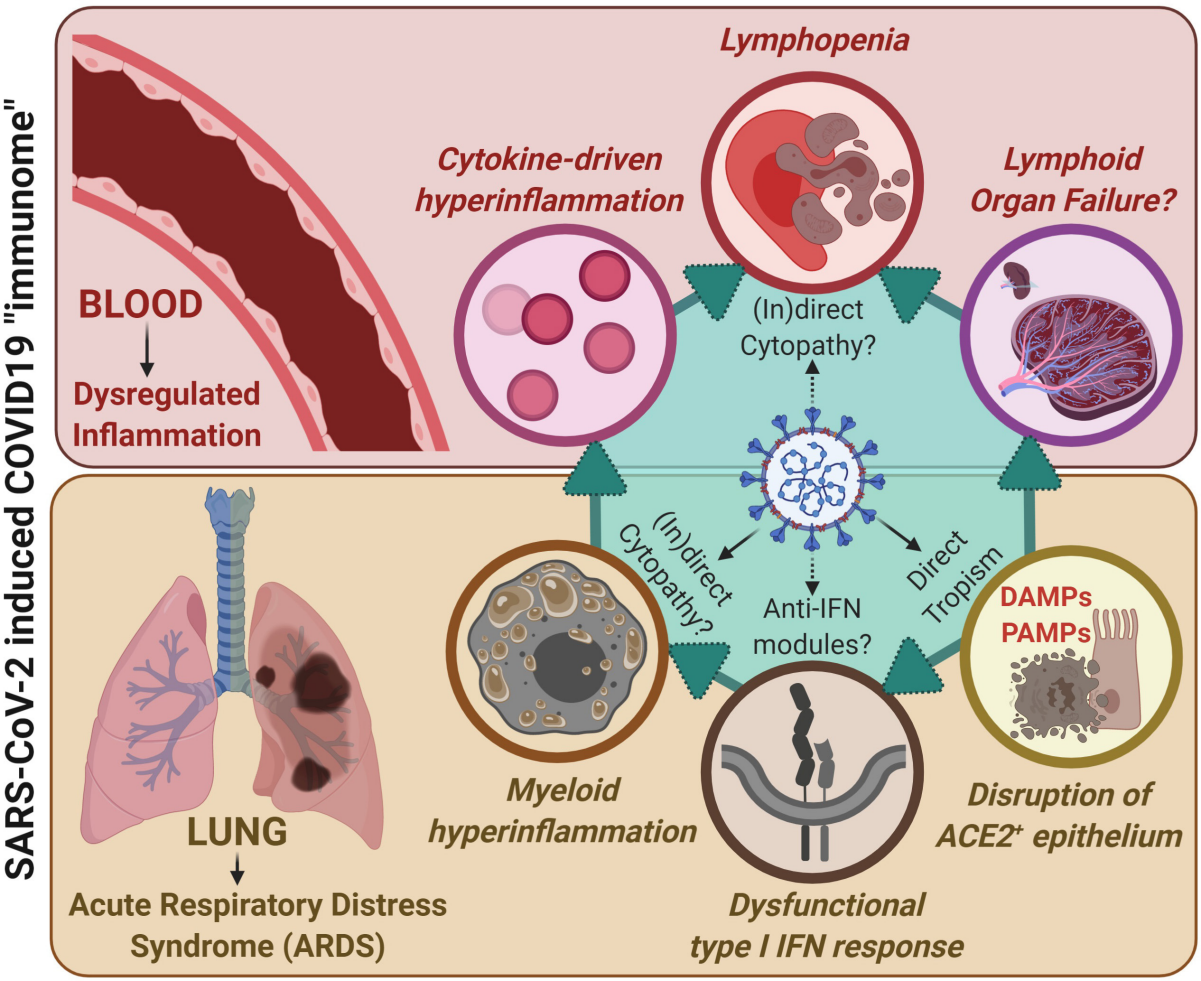
A unified COVID-19 “immunome” model integrating lung-associated pathophysiology with systemic immunopathology, together accounting for the SARS-CoV-2/COVID19 immunological paradigm. SARS-CoV-2 exhibits increased (direct) tropism toward ACE2+ epithelial cells within the lungs and upper-airways that, due to viral replication, ultimately results in epithelial cell death and epithelium disruption. This is paralleled by disruption of type I interferon (IFN) responses within infected cells, possibly due to direct interference by SARS-CoV-2 derived anti-IFN modules. This is accompanied by release of damage-associated molecular patterns (DAMPs; coming from dying/dead epithelial cells) as well as pathogen-associated molecular patterns (PAMPs; coming from viral genetic material and immunogenic proteins). These events, together with the above viral pathogenic events, fuel dysregulated myeloid hyperinflammation. In addition, through as-yet-unclear mechanisms, SARS-CoV-2 may also exert direct or indirect cytopathic effects on myeloid immune cells, thereby further facilitating immune dysregulation. These events together characterize the COVID-19-associated ARDS phenotype. This ARDS can: (I) on one hand, facilitate systemic cytokine-driven hyperinflammation; yet (II) on the other hand, stress the lymphoid organs by demanding increased recruitment of immune cells for resolution of inflammation. Such prolonged immunological stress accompanied by cytokine-based inflammation facilitates lymphopenia, typically observed in COVID-19 patients (and may also cause lymphoid organ failure if this stressful situation prolongs, as seen in some critically ill patients). Herein, there is some evidence that SARS-CoV-2 may exert (in)direct cytopathic effects against lymphocytes thereby further fuelling lymphopenia. Together these processes define the overall immunological phenotypes or inclusive phenome (i.e., “immunome”) of COVID-19.

Coronavirus disease-2019 (COVID-19), caused by severe acute respiratory syndrome coronavirus-2 (SARS-CoV-2), has created an unprecedented global health crisis. Because of its recent emergence, there is paucity of knowledge on viral pathogenesis and host immune responses to SARS-CoV-2. Most of the currently published research has explored either the host immune response (*i.e.*, host immunopathology) or SARS-CoV-2 lung epithelium tropism (*i.e*., viral pathogenesis) (1–3). Acute respiratory distress syndrome (ARDS), cytokine storm, lymphopenia and exhausted lymphocytes (in particular T cells) are the most proposed immunopathological phenotypes, while cellular injury due to virus-host interactions and interferon (IFN) dysregulation are well-described for viral pathogenesis (1, 3). Despite these insights, COVID-19 remains a “moving immunological concept” due to frequent disagreements in literature over some major immunological characteristics. In this opinion, we organize the current knowledge and integrate some hypotheses and anticipated challenges into a broad COVID-19 “immunome” paradigm, i.e., the entirety of processes defining the overall immunological phenotypes or inclusive phenome (see legend of Figure 1), that is exploitable for guiding future mechanistic biomarker-mining in longitudinal clinical samples collected from COVID-19 patients.

## Covid-19 Immunopathology: “A Moving Concept”

The upper respiratory system is the main port-of-entry for SARS-CoV-2. The infection can eventually progress into hyperinflammation in the lungs, and in severe cases into respiratory insufficiency and ARDS (2) - see overview in Figure 1. This negative clinical evolution is almost always accompanied by a dysregulated local and systemic immune response, comprising hyperactive circulating as well as lung-infiltrating myeloid immune cells (especially monocytes, macrophages and neutrophils). Their activation is triggered by virus-associated or induced pathogen- and damage-associated molecular patterns (i.e., PAMPs and DAMPs) and results in an excessive release of chemokines and inflammatory cytokines (1, 2, 4, 5). It has been proposed that this excessive production of cytokines is reminiscent of a typical cytokine-release syndrome (CRS), which in COVID-19 is coupled to reduced IFN production and a defective antiviral defense (3, 6, 7). However, in-depth insights into the innate immune hyperactivity in COVID-19 are still lacking. Moreover, in most COVID-19 patients that require hospitalization, there is a striking lymphopenia that correlates with clinical severity and involves all lymphocyte subpopulations (1–3). In addition lymphocyte subset alterations have been observed and indeed, CD8 T- and B-cell quantities as well as CD4/CD8 ratio seem to act as independent predictors of COVID-19 survival (8). A major question, however, remains how myeloid hyperactivation and T-/B-cell dysfunction contribute to SARS-CoV-2 immunopathology. Also, can SARS-CoV-2 exert direct and indirect cytopathic effects on immune cells? For this to happen, an alternative SARS-CoV-2 “entry mechanism,” other than the one for respiratory epithelial cells involving viral spike-glycoproteins and angiotensin-converting enzyme 2 (ACE2) receptor interaction, must exist for immune cells that do not strongly express ACE2 receptors (2, 3, 9). In this regard, on the one hand SARS-CoV-2 may gain cellular entry via the CD147 receptor that is widely expressed on immune cells, accounting for direct immunocytopathic effects (10, 11). On the other hand, in analogy with e.g., dengue and influenza viruses (12), an indirect immunocytopathy may exist through antibody-dependent enhancement (ADE) mechanisms, in which non-neutralizing virus-specific antibodies may facilitate viral entry into myeloid cells via Fc receptors-mediated phagocytosis or efferocytosis (13). However, the uptake of viral particles via such mechanisms will be suboptimal, leading to inflammatory activation of infected cells but not orderly viral degradation (12). This hypothesis is intriguing because it has been observed that a small fraction of COVID-19 patients that mount early (possibly low-quality) antibody responses against SARS-CoV-2, tend to suffer from more severe symptoms (12). Moreover, COVID-19 patients with agammaglobulinemia and negligible B-cells presence may have a milder clinical course of COVID-19 (14). Unfortunately, little is known on the exact interactions between SARS-CoV-2 on immune cells, thereby presenting an additional important knowledge gap that needs to be addressed urgently.

## SARS-CoV-2: From Cellular Pathogenesis to Immunological Consequences

Another level of immune-dysregulation during COVID-19 is positioned at the antiviral immunity induced by SARS-CoV-2-infected cells. A normal antiviral response involves a sustained type I/III interferon (IFN) cytokine-response that activates IFN-stimulated-genes (ISGs), which then orchestrate a multi-parametric program of antiviral defenses (2, 15). SARS-CoV-2 can disrupt these protective inflammatory reactions by blunting type I/III IFN-cytokines, while increasing other pro-inflammatory cytokines (6, 7). This imbalanced cytokine response can impede efficient inhibition of viral replication and viral clearance (7). Whole blood RNA-profiling indeed showed that critically-ill COVID-19 patients initiate inefficient type I IFNs-responses, have reduced ISGs and persistent (blood-borne) viral load, and show an exacerbated inflammatory response driven by IL6, TNF, and IL1β/IL1RA (6). Importantly, these dysregulations are capable of driving immunocytopathic or immune cell death phenomena e.g., apoptosis/necroptosis in lymphocytes or pyroptosis and NETosis in neutrophils, thereby further worsening inflammation (4, 16). Moreover, human population-related genetic variation studies have shown that evolution-driven natural selection has targeted the human IFNs pathway with variable (purifying) selection or (geographically-restricted) positive selection (17). Thus, various human populations may differ widely in terms of their anti-viral IFNs pathway mechanics. And this may further account for heterogeneity in COVID-19 symptoms and progression. Together this again illustrates the importance of connecting characteristics of SARS-CoV-2 viral pathogenesis with host immunopathological characteristics to fully unravel COVID-19 pathophysiology.

## COVID-19 “Immunome”: How to Conceptualize and Study A Unified Paradigm?

Successful COVID-19 patient management and therapeutic intervention will likely relies on comprehensively integrating all knowledge on SARS-CoV2-tropism and COVID-19-immunopathology into a single unified “immunome”-paradigm. The integration of currently published evidence (1–3) and preliminary working-hypotheses (6, 7, 10, 12, 14) indicates a spatio-temporal immune-dysregulation during COVID-19. In particular, we present a model of two cross-talking immunopathologies (Figure 1), which may both culminate into indirect and/or direct immune-cytopathic effects: *(i)* a viral tropism-based lung infection (VLI) leading to cytokine-driven hyperinflammation coupled with dysfunctional IFN-responses that impede viral clearance at the entry site (upper respiratory airways/lungs); and *(ii)* collateral systemic immune-dysregulation (CSID) resulting from an excessive and ineffective triggering of myeloid immune cells (3, 18–21). The latter may further drive an uncoordinated cytokine response compromising proficient lymphocytic and NK cell functioning (22). Moreover, we propose that when VLI is excessive and progressing in a very short time frame, this may rapidly “titrate” several important immune cells away from systemic presence for resolution of the lung-associated inflammation, thereby stressing the lymphoid organs (3). If this extreme immunological imbalance continues without resolution of the lung inflammation, it may pave the way for a depletion-like phenotype for certain immune cells and facilitate CSID (23). We hypothesize that these combined immunopathologies may further hamper a correct antiviral (protective) T-/B-cell response through mechanisms that have not been comprehensively elucidated. The inter-individual determinants and risk factors for various inflammatory pathways are currently unknown, and more knowledge is necessary to refine our model. Clearly, it is important to rapidly understand the cause-effect components of the COVID-19-associated inflammatory cycle: *i.e.*, it is essential to differentiate whether genotype-specific differences in the (innate) immune response are causing COVID-19 pathology or whether the immunological phenotype is a consequence of its rapidly mounting pressure on the host immune-inflammatory system. In the end, a combination of both intrinsic host-related immune features and an excessive persistent viral attack on the immune system can be mediating the pathological features of COVID-19.

Confirming this model on a mechanistic level would require a high degree of harmony between methodological initiatives and clinical study-design, starting with the collection of dedicated and hard-to-get (invasive) patient samples. These should include samples of different tissues (lung, heart, spleen, liver, bone marrow and lymph nodes upon autopsy, and bronchoalveolar lavage), and longitudinal blood samples. For instance, there is a huge scarcity of high quality autopsy analyses in COVID-19 deceased patients, thereby severely compromising our understanding of this complex disease in the terminal time-frame (24). In addition to basic immunoprofiling technologies (flow cytometry/cytokine profiling), single cell-transcriptomics should be included in the workflow. This will allow in-depth dissection of the fundamental immune cell-states: i.e., immune cell differentiation and functional trajectories as well as discrimination of virus infected or uninfected immune cells. Such integrated knowledge will play a major role in understanding COVID-19 pathophysiology and will serve as input for dedicated drug discovery pipelines. For instance, a recent clinical trial that accounts for both viral pathogenesis and immunopathology, by administrating triple-therapy consisting of IFN-1β, lopinavir-ritonavir and ribavirin, alleviated symptoms, suppressed IL-6 levels and shortened duration of viral-shedding and hospital stay in mild-to-moderate COVID-19 patients (9). Similar multi-level therapeutic solutions are required for severely ill patients and need to consider the multi-parametric nature of COVID-19. Thus, a COVID-19 “immunome” driven approach can lead to better patient management and therapeutic decisions.

## Author Contributions

All authors contributed toward conceptualization of ideas presented in this manuscript through intensive discussions and brain-storming. EW, KT, CW, and FB helped in writing of the manuscript. JW, ST, and AG provided overall supervision and guidance. Overall, this represents the assimilated opinion of our CONTAGIOUS consortium currently working on immuno-profiling of COVID19 patients.

## Conflict of Interest

The authors declare that the research was conducted in the absence of any commercial or financial relationships that could be construed as a potential conflict of interest.
